# Supplementary material for: Preoperative High C-Reactive Protein to Albumin Ratio Predicts Short- and Long-Term Postoperative Outcomes in Elderly Gastric Cancer Patients
Source: Cancers (Basel). 2024 Jan 31;16(3):616. doi: 10.3390/cancers16030616 (PMC10854578; doi:10.3390/cancers16030616)
Supplement: Supplementary file 1 [file cancers-16-00616-s001.zip › cancers-2803545-supplementary.pdf]

Supplemental Table S1. Comparison of the characteristics between the CAR high and low group.

	CAR-high (≥0.024) group (n = 215)	CAR-low (<0.024) group (n = 356)	p-value
Age	71 (21-89)	68 (33-89)	**< 0.001**
Sex			0.135
Male	156 (72.6)	237 (66.6)	
Female	59 (27.4)	119 (33.4)	
BMI	22.5 (15.2-32.9)	22.0 (14.5-31.2)	0.404
ASA-PS			**< 0.001**
1	11 (5.1)	50 (14.0)	
2	176 (81.9)	294 (82.6)	
3	28 (13.0)	12 (3.4)	
Charlson Comorbidity Index*			**< 0.001**
Low: 0	95 (44.2)	231 (64.9)	
Medium: 1-2	85 (39.5)	102 (28.7)	
High: 3-4	28 (13.0)	19 (5.3)	
Very high: ≥5	7 (3.3)	4 (1.1)	
Location of tumor			0.788
Upper	83 (38.6)	138 (38.8)	
Middle	75 (34.9)	132 (37.1)	
Low	57 (26.5)	86 (24.1)	
Histopathological type**			**< 0.001**
Differentiated	127 (59.1)	154 (43.3)	
Undifferentiated	88 (40.9)	202 (56.7)	
Depth of tumor***			**0.002**
T1a-1b	137 (63.7)	256 (71.9)	
T2	19 (8.8)	46 (12.9)	
T3	34 (15.8)	26 (7.3)	
T4a-4b	25 (11.6)	28 (7.9)	
Lymph node metastasis***			**0.027**
N0	148 (79.2)	282 (79.2)	
N1	28 (13.0)	33 (9.3)	
N2	22 (10.2)	18 (5.1)	
N3	17 (7.9)	23 (6.5)	
Pathological stage***			**0.004**
I	145 (67.4)	284 (79.8)	
II	29 (13.5)	32 (9.0)	
III	41 (19.1)	40 (11.2)	

Variables in bold are statistically significant (P < 0.05). Categorical variables were described using frequencies and percentages and continuous variables were described using median and range.

* Charlson Comorbidity Index were defined by only comorbidities without adjusting age. ** Differentiated type and undifferentiated type involved papillary carcinoma, tubular adenocarcinoma and poorly differentiated adenocarcinoma, mucinous adenocarcinoma, signet-ring cell carcinoma, respectively. *** According

to the 15th edition of the Japanese Classification of Gastric Carcinoma.

Abbreviation: ASA-PS, American society of anesthesiologists physical status; BMI, body mass index; CAR, C-reactive protein/albumin ratio; CD, Clavien-Dindo classification
